# The Interaction between AID and CIB1 Is Nonessential for Antibody Gene Diversification by Gene Conversion or Class Switch Recombination

**DOI:** 10.1371/journal.pone.0011660

**Published:** 2010-07-20

**Authors:** Zachary L. Demorest, Donna A. MacDuff, William L. Brown, Scott G. Morham, Leslie V. Parise, Reuben S. Harris

**Affiliations:** 1 Department of Biochemistry, Molecular Biology and Biophysics, Institute for Molecular Virology, Beckman Center for Genome Engineering, University of Minnesota, Minneapolis, Minnesota, United States of America; 2 Myriad Pharmaceuticals, Salt Lake City, Utah, United States of America; 3 Department of Biochemistry and Biophysics, University of North Carolina, Chapel Hill, North Carolina, United States of America; Yale Medical School, United States of America

## Abstract

Activation-induced deaminase (AID) initiates somatic hypermutation, gene conversion and class switch recombination by deaminating variable and switch region DNA cytidines to uridines. AID is predominantly cytoplasmic and must enter the nuclear compartment to initiate these distinct antibody gene diversification reactions. Nuclear AID is relatively short-lived, as it is efficiently exported by a CRM1-dependent mechanism and it is susceptible to proteasome-dependent degradation. To help shed light on mechanisms of post-translational regulation, a yeast-based screen was performed to identify AID-interacting proteins. The calcium and integrin binding protein CIB1 was identified by sequencing and the interaction was confirmed by immunoprecipitation experiments. The AID/CIB1 resisted DNase and RNase treatment, and it is therefore unlikely to be mediated by nucleic acid. The requirement for CIB1 in AID-mediated antibody gene diversification reactions was assessed in CIB1-deficient DT40 cells and in knockout mice, but immunoglobulin gene conversion and class switch recombination appeared normal. The DT40 system was also used to show that CIB1 over-expression has no effect on gene conversion and that AID-EGFP subcellular localization is normal. These combined data demonstrate that CIB1 is not required for AID to mediate antibody gene diversification processes. It remains possible that CIB1 has an alternative, a redundant or a subtle non-limiting regulatory role in AID biology.

## Introduction

Following V(D)J recombination, the expressed antibody repertoire in vertebrate B lymphocytes gains additional diversity through the processes of immunoglobulin (Ig) gene conversion (IGC), somatic hypermutation (SHM), and class-switch recombination (CSR) (reviewed by [1]–[3]). Most vertebrates use SHM to diversify variable (V) region sequences, but a few species, such as birds and rabbits, use a template-dependent IGC mechanism to generate antigen-binding diversity and improve antibody affinity. All vertebrates use CSR to irreversibly change the constant (C) region of the heavy chain Ig gene, which dictates the antibody tissue distribution and function.

All three of these antibody gene diversification reactions are initiated at the DNA level by activation-induced cytidine deaminase (AID)-catalyzed deoxy-cytidine to deoxy-uridine editing ([4]; reviewed by [1]–[3]). Once these lesions occur in Ig gene DNA, ubiquitious DNA ‘repair’ enzymes catalyze further reactions that ultimately result in the distinct outcomes described above. For instance, both the uracil DNA glycosylase UNG2 and the mismatch recognition proteins MSH2/6 help process AID-catalyzed DNA uridines [5]–[7].

Several lines of evidence indicate that AID is subject to numerous levels of post-translational regulation. First, AID is predominantly cytoplasmic, and recent data suggest that this may occur through an active retention mechanism [8]–[10]. Second, AID is actively imported into the nuclear compartment, a step that is undoubtedly required for its essential role in antibody gene diversification [10], [11]. Third, AID is exported from the nuclear compartment by virtue of a carboxy-terminal leucine-rich motif, which is bound by CRM1 and necessary for export [9]–[13]. Fourth, AID is susceptible to post-translational regulation by ubiquitination and phosphorylation [14]–[20]. The responsible E3 ubiquitin ligation complex has yet to be identified, but protein kinase A (PKA) has been shown to phosphorylate AID [14], . Finally, phosphorylation enhances the interaction between AID and replication protein A, which may be an important DNA level co-factor [21]. These studies combine to indicate that multiple cellular proteins regulate AID, and several of these have yet to be identified.

To gain further insights into the proteins that regulate AID, we used high-throughput yeast two-hybrid screening to identify interacting proteins. We found that AID interacts with the calcium and integrin binding protein 1 (CIB1), a 22 kDa regulatory protein that is broadly expressed, comprised of four EF hand motifs (two of which bind calcium), and required for spermatogenesis in mice [22], [23]. CIB1 is a provocative AID-interacting protein, because it could potentially link B cell receptor signaling, activation through calcium fluctuations, and the germinal center specific antibody gene diversification reactions. The requirement for CIB1 in AID-mediated processes was examined in two model systems for antibody gene diversification, the chicken B cell line DT40 and murine B lymphocytes.

## Results

### The AID-CIB1 interaction

A large scale, Gal4-based yeast two-hybrid screen was conducted as described [24]. A bait plasmid expressing human AID residues 56–198 was used to screen a tongue and tonsil cDNA library. DNA sequencing was used to identify candidate interactors, and false positives were eliminated by cross checking literature, public databases, and unpublished data. Only one positive hit resisted triage: residues 1–191 of the calcium and integrin binding protein 1 (CIB1). Other reported AID-interaction proteins, DNA-PKcs [25], MDM2 [26], RPA [21], PKA [14], [18], and CTNNBL1 [27], were not recovered.

**Figure 1:**
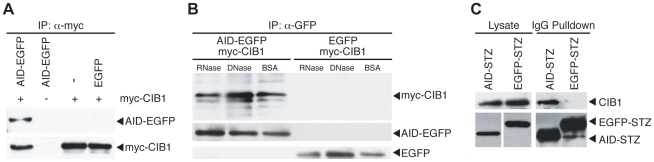
AID interacts with the calcium and integrin binding protein 1. **A**. myc-CIB1 co-IPs AID-EGFP but not EGFP only (left and right lanes, respectively). A myc-specific monoclonal antibody was used to precipitate complexes, and AID-GFP was detected with an α-GFP polyclonal antibody. **B**. AID-EGFP co-IPs myc-CIB1 in a DNase I- and RNase A-resistant manner. An α-GFP monoclonal antibody was used to precipitate AID-GFP, and myc-CIB1 was detected with an α-myc monoclonal antibody. **C**. AID-STZ pulls down endogenous CIB1 from HEK-293T cell extracts. IgG Sepharose was used to pull-down STZ complexes, and CIB1 was detected using an α-CIB1 polyclonal antibody. AID-STZ and GFP-STZ were detected with an α-strep antibody. A low level of non-specific background was observed in the vicinity of AID-STZ. For cell lysate (input) control blots, two panels are shown because GFP-STZ is expressed over 100-fold better than AID-STZ. A quantification of the input versus pull-down signal indicated that 1% of cellular CIB1 can be pulled-down with AID complexes when IgG sepharose beads are limiting.

To confirm the yeast two-hybrid result, we co-expressed AID-EGFP and myc-CIB1 in HEK-293T cells and asked whether the two proteins interacted by co-immunoprecipitation (co-IP). These experiments showed that myc-CIB1 was able to co-IP AID-EGFP (Fig. 1A). In contrast, myc-CIB1 did not co-IP an EGFP control.

AID can bind single-stranded nucleic acids, including both DNA and RNA [28]. AID homologs such as APOBEC3G also bind RNA, and this interaction enables indirect associations with many different cellular proteins [29]–[33]. To ask whether the AID-CIB1 interaction occurs through a nucleic acid bridge, co-IP experiments were repeated in the presence of DNase or RNase. An α-GFP (AID) IP resulted in strong myc-CIB1 immunoblot signals, which were not diminished by incubating extracts with either DNase I or RNase A (Fig. 1B). These data strongly suggested that the interaction between AID and CIB1 occurs independently of a nucleic acid bridge.

To further substantiate the AID-CIB1 interaction, a pull-down experiment was performed with a tandem affinity purification construct AID-STZ (Materials and Methods). This construct, or an EGFP-STZ control, was transfected into HEK-293T cells, expressed for 48 hours, and ultimately pulled-down with IgG Sepharose, which binds the Z domain of Protein A. Immunoblotting with a CIB1-specific monoclonal antibody revealed that endogenous CIB1 associates with AID-STZ but not with EGFP-STZ, which was even more highly expressed (Fig. 1C).

### CIB1 over-expression in DT40

**Figure 2:**
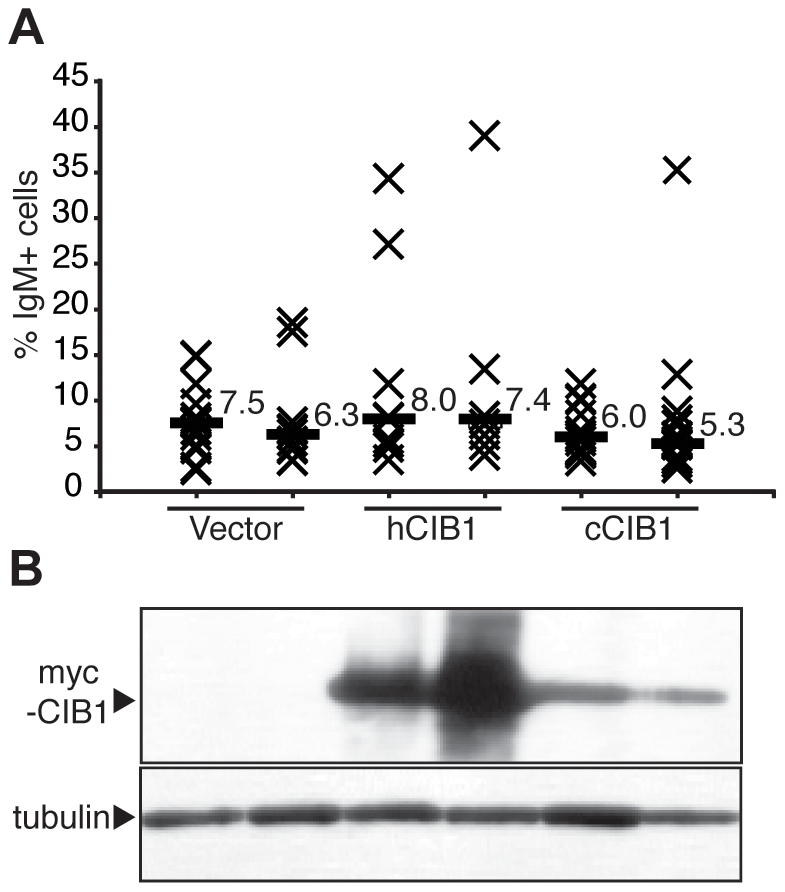
CIB1 over-expression in DT40. **A**. An IGC fluctuation analysis showing the percentage of surface Ig-positive cells in subclone cultures over-expressing human (h) or chicken (c) CIB1. Each X represents data from an individual subclone and the labeled horizontal bars report the medians for each data set. **B**. CIB1 over-expression confirmed by immunoblotting. Loading was controlled by stripping and re-probing the blot with an α-tubulin antibody.

To begin to test whether CIB1 regulates AID-dependent antibody gene diversification reactions, we over-expressed both human and chicken CIB1 proteins in the chicken B cell line DT40. This cell line constitutes a good model system for studying IGC, because the reversion of an Ig frameshift mutation is AID-dependent and can be monitored at the cellular level by flow cytometry of surface Ig positive cells [34], [35]. CIB1 over-expressing lines were established and subjected to limiting dilution, and 8–12 of the resulting subclones were cultured continuously for 6 weeks. The median percentage of surface Ig positive cells among the control vector transfected subclones was 7.5%, and the medians of the CIB1 over-expressing subclones were not significantly different (Fig. 2). These data indicated that CIB1 protein levels are not limiting in DT40 for gene conversion at the antibody locus.

### Immunoglobulin gene conversion in CIB1 knockout DT40

**Figure 3:**
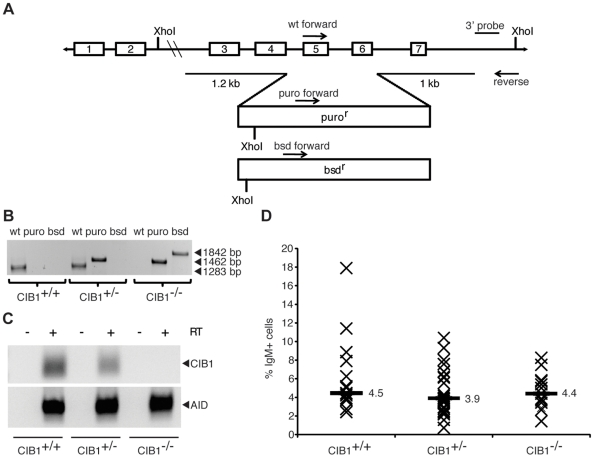
CIB1 is dispensable for immunoglobulin gene conversion in DT40. **A**. Schematic showing the constructs used to replace exons 5 and 6 of *CIB1* with the indicated drug resistance cassettes. The positions of the allele-specific PCR primers for genotyping and the XhoI sites and the position of the 3′ external probe used for Southern blot analysis are shown. **B**. An agarose gel image showing the allele-specific PCR products from CIB1+/+, +/−, and −/− cell lines. **C**. An agarose gel image of CIB1-specific RT-PCR products from CIB1+/+, +/−, and −/− cell lines. AID-specific reactions were used to demonstrate the integrity of each cDNA preparation. **D**. An IGC fluctuation analysis showing the percentage of surface Ig-positive cells in subclone cultures of the indicated genotype. Each X represents data from an individual subclone and the labeled horizontal bars report the medians for each data set.

To determine whether CIB1 is required for IGC in DT40, serial gene targeting was used to delete exons 5 and 6 of the *CIB1* gene (Fig. 3A). A linearized targeting vector encoding the puromycin-resistance gene was transfected into a surface Ig-negative DT40 line and resistant colonies were isolated. Gene targeting events were identified by PCR (Figs. 3A & B). *CIB1* heterozygous lines were subjected to a second round of gene targeting with a blasticidin resistance construct. CIB1 null lines were identified by allele-specific PCR reactions and confirmed by reverse-transcription PCR and Southern blot analyses (Figs. 3B & C and data not shown). The CIB1 defective cells showed no obvious growth defects or cellular abnormalities demonstrating that this protein is not essential in DT40 cells.

To ask whether AID-dependent IGC requires CIB1, surface Ig-negative CIB1^+/+^, CIB1^+/−^, and CIB1^−/−^ cell lines were subcloned by limiting dilution, maintained in culture for 6 weeks, labeled with α-Ig antibody, and quantified by flow cytometry. We found that the median frequency of surface Ig-positive subclones was nearly identical for CIB1^+/+^, CIB1^+/−^, and CIB1^−/−^ cell lines, 4.5%, 3.9%, and 4.4%, respectively (Fig. 3D). These data provided an unambiguous demonstration that CIB1 is not required for IGC in DT40.

### AID localization in CIB1 knockout DT40

**Figure 4:**
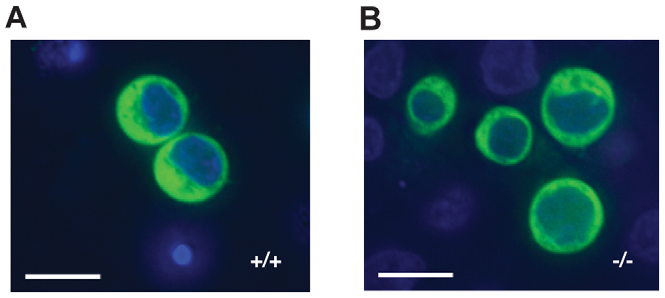
AID localization in CIB^−/−^ DT40 cells. **A**. AID-EGFP localization in CIB1^+/+^ DT40. **B**. AID-EGFP localization in CIB1^−/−^ DT40. Images were taken using a 40× objective and the scale bars indicate 10 µm.

Another property of AID is that it localizes predominantly to the cytoplasm of cells (*e.g*., [8]–[10], [36]). To ask whether CIB1 is required for this process, we stably transduced wildtype and CIB1-deficient DT40 cells with an AID-EGFP expression construct and used fluorescent microscopy to image the resulting localization patterns (Fig. 4). The cytoplasmic localization of AID-EGFP was indistinguishable in wildtype and CIB1-deficient cells indicating that CIB1 is also dispensable for this property.

### Isotype switching in CIB1 knockout mice

**Figure 5:**
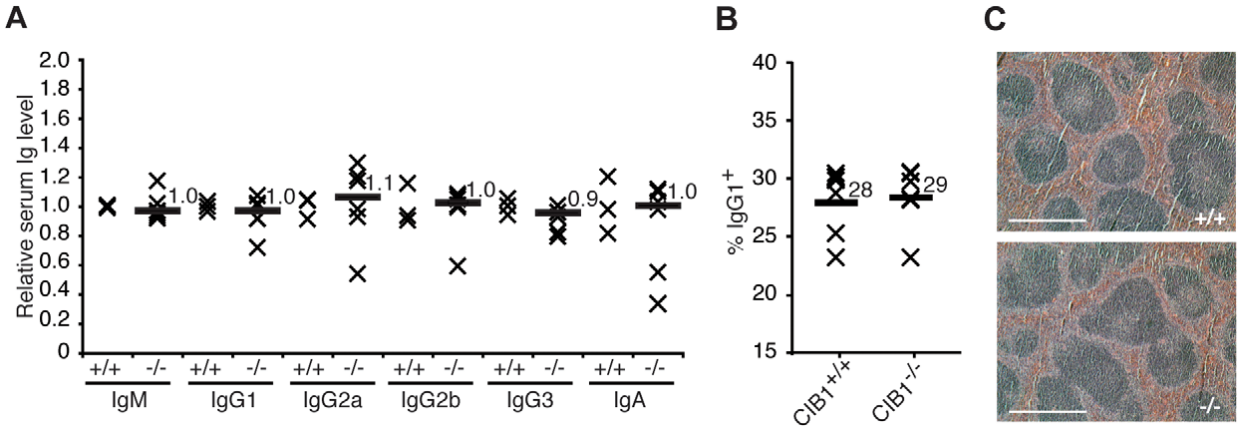
CIB1 is dispensable for CSR in mice. **A**. Relative levels of each antibody isotype in sera from CIB1^+/+^ or CIB1^−/−^ mice as measured by ELISA. The CIB1^−/−^ data were normalized to the mean antibody levels in sera from wildtype (WT) littermates (arbitrarily set to 1 for comparison). Each X represents data from an independent animal and the horizontal bars and labels report the median values (n = 3 for CIB^+/+^ and n = 6 for CIB^−/−^). **B**. IgM to IgG1 CSR *ex vivo*. B-cells were isolated from the spleens of CIB1^+/+^ or CIB1^−/−^ mice, cultured for 4 days in the presence of LPS and IL-4, and analyzed by α-IgG1-PE labeling and flow cytometry. Each X represents data from an independent animal and the horizontal bars and labels report the median values. **C**. Images of hematoxylin and eosin stained sections of spleen isolated from CIB1^+/+^ and CIB1^−/−^ mice. Scale bars indicate 500 µm.

To evaluate the potential contribution of CIB1 to another AID-dependent process in another model organism, we examined the CSR phenotype of CIB1-deficient mice [23]. First, we measured the abundance of alternative antibody isotypes in sera from 3 wildtype and 6 CIB1-deficient mice by ELISA (Fig. 5A). Normal levels of IgM, IgG1, IgG2a, IgG2b, IgG3, and IgA were observed indicating that a CIB1 deficiency does not result in a gross CSR defect.

However, minor deficiencies in CSR can be readily eclipsed by antigen driven clonal expansions of alternative isotype expressing B lymphocytes. We therefore next examined the capacity of wildtype and CIB1-deficient primary B lymphocytes to undergo CSR *ex vivo* in response to lipopolysacharride (LPS) and interleukin-4 (IL-4) treatment. This stimulatory regimen drives CSR from IgM to IgG1, which is easily quantified by anti-IgG1 staining and flow cytometry (*e.g*., [5]). After 4 days in culture, 28% of the wildtype cells expressed surface IgG1 and near-identical results were obtained with CIB1-deficient B lymphocytes (Fig. 5B). These data combined to demonstrate that CIB1 is not required for CSR in mice or in murine B lymphocytes *ex vivo*.

### Germinal center morphology in CIB1 knockout mice

AID-deficient mice have enlarged germinal centers and spleenomegaly [37]. To ask whether the CIB1 interaction with AID may be important for proper development *in vivo*, we compared the morphology of the spleens of wildtype and CIB1-deficient mice. Spleens were removed, sectioned, and stained with hematoxylin and eosin. CIB1-deficient mice were found to have a normal spleen and germinal center morphology (Fig. 5C).

## Discussion

AID-mediated antibody diversification reactions are conserved processes in vertebrates. However, the precise mechanisms that regulate AID remain to be fully understood. We identified the calcium and integrin binding protein 1 (CIB1) as a strong AID-interacting protein. This protein is an attractive regulatory candidate because calcium signaling is an integral part of antigen-dependent adaptive immune responses. However, the complete ablation of CIB1 in DT40 or mice demonstrated that this protein is dispensable for both IGC and CSR in these organisms (*i.e*., CIB1 is not an antibody diversification co-factor). Moreover, the lack of any detectable antibody diversification phenotype also indicated that CIB1 is unlikely to be a critical positive or negative regulator of AID. This point was further underscored by the lack of an Ig diversification phenotype in CIB1 over-expressing DT40.

CIB1 is however important for other functions in vertebrates. It is a nonenzymatic regulatory molecule that binds an array of target proteins and modulates their activity. For example, CIB1 activates the serine/threonine kinase p21-activated kinase (PAK1) and thereby modulates the migratory and proliferative capacities of cells [38]. It inhibits polo-like kinase 3 (PLK3), which may slow the G1 phase of the cell cycle [39], and it inhibits apoptosis signal-regulating kinase (ASK1), which negatively regulates the stress-activated MAP kinases JNK and p38 [40]. The CIB1 knockout mouse develops near normally under non-stressed conditions except that the CIB1−/− male is sterile [23]. This is most likely due to a disrupted progression of the haploid phase of spermatogenesis. Significantly slowed proliferation of mouse embryo fibroblasts was also noted. While the exact cause of the disrupted haploid phase is unclear, it may be related to elevated levels of the cell cycle regulator CDC2/CDK1.

Since the interaction between AID and CIB1 is robust, we were surprised that antibody gene diversification reactions are not obviously affected. This could be due to a functional overlap between CIB1 and the related calcium and integrin binding proteins, CIB2 and CIB3, which share over 60% amino acid similarity [22], [41]. However, AID may have other roles in the biology of vertebrates, such as the provision of innate immunity to retrotransposition [36], [42]–[44], the restriction of foreign DNA [45], or the epigenetic reprogramming of stem cells [46],[47]. Future studies will be necessary to address the role of CIB1 in these potentially interesting alternatives.

## Materials and Methods

### Ethics Statement

All experimental procedures for working with mice, described in full below, were approved by the University of Minnesota Institutional Animal Care and Use Committee (IACUC Protocol Number: 0703A04446).

### DNA constructs

The parental myc expression vector encoding 6 copies of an N-terminal myc tag was a generous gift from Dr. Shigeki Miyamoto (University of Wisconsin) [48]. Human *CIB1* cDNA was PCR amplified from a spleen cDNA library (Invitrogen) using primers 5′-GAA-TTC-TGA-TGG-GGG-GCT-CGG-GCA-GTC-GC and 5′-CTC-GAG-TCA-CAG-GAC-AAT-CTT-AAA-GGA-GCT-G, digested with EcoRI/XhoI and ligated into p6xmyc to generate p6xmyc-hCIB1. Chicken *CIB1* cDNA was PCR amplified from DT40 cDNA using primers 5′-GAA-TTC-TCA-TGG-GGG-GCT-CCA-GCA-GTC-TGC and 5′-CTC-GAG-TCA-CAG-GAC-AAT-CTT-GAA-GG, digested with EcoRI/XhoI, and ligated into p6xmyc to generate p6xmyc-cCIB1.

Human AID cDNA [26] was PCR amplified using primers 5′-TAA-TAC-GAC-TCA-CTA-TAG-GG and 5′-GTC-GAC-AAG-TCC-CAA-AGT-ACG-AAA-TGC, digested with HindIII/SalI, and ligated into similarly cut pEGFP-N3 (Clontech). The lentiviral transduction vector pCSII-AID-EGFP was generated by directly subcloning AID-EGFP from pEGFP-N3-AID into pCSII-EF-MCS [49] using the XhoI/NotI sites. The lentiviral packaging and helper plasmids pMDG and ΔNRF were provided by Dr. Nik Somia (University of Minnesota) [50], [51].

The tandem affinity purification constructs, pAID-STZ and pEGFP-STZ (Strep, TEV, Z domain), were made as follows. First, a Tobacco Etch Virus (TEV) protease cleavage site (ENLYFQG) was cloned into the EcoRI/XmaI sites of pBluescript II KS+ (Stratagene). Second, two tandem copies of the IgG binding Z domain of protein A were amplified by PCR from pRAV-flag [52] using primers 5′-CCC-GGG-ATG-AGG-TTA-ACC-ATG-GCG-CAA and 5′-GAG-CTC-TCT-AGA-TTA-CGC-GTC-TAC-TTT-CGG-CGC-CTG and cloned into SacI/SmaI sites of pBluescript-TEV. Third, a XhoI-NotI-EcoRI linker was added by ligating annealed primers 5′-TCG-AGA-GCG-GCC-GCA-TG and 5′-AAT-TCA-TGC-GGC-CGC-TC into the XhoI/EcoRI sites. Fourth, a Streptavidin tag was added by ligating annealed oligonucleotides 5′-GGC-CGC-ATG-GCT-AGC-TGG-AGC-CAC-CCG-CAG-TTC-GAA-AAA-GGC-GCC and 5′-GGC-CGG-CGC-CTT-TTT-CGA-ACT-GCG-GGT-GGC-TCC-AGC-TAG-CCA-TGC into the NotI site. Fifth, Strep-TEV-ZZ was subcloned into the XhoI/XbaI sites of pcDNA3.1(Invitrogen). Finally human AID or EGFP were subcloned into KpnI/SalI sites of this construct to generate pAID-STZ and pEGFP-STZ.

The chicken *CIB1* targeting vectors were generated by amplifying targeting arms from DT40 genomic DNA using primers 5′-GGC-CCG-TTT-TCG-TCC-CCC-GGA and 5′-ACG-GCA-CCT-CCG-TGC-GGG-AGC (1.2 kb left arm) and 5′-TCC-AGC-AGC-ATA-AGG-GGT-CCC and 5′-CTG-CAC-AGA-GCT-CGT-TCC-CCA (1.0 kb right arm). These PCR products were cloned into pCR-BluntII-TOPO (Invitrogen) and subcloned into pBluescript II KS+ (Stratagene) using NotI/BamHI (left arm) and EcoRI/HindIII (right arm). The puromycin or blasticidin resistance cassettes were then subcloned from bML4 or bML5 [34] into the BamHI site between the homology arms.

### Immunoprecipitation experiments

HEK-293T cells [32], [38] were transfected with 5 µg of plasmid pEGFP-N3-AID or pEGFP-N3 and p6xmyc-CIB1 in a 10 cm dish (∼60% confluent) using a ratio of 1 µg DNA to 3 µl FuGene6 (Roche). 48 hrs post-transfection, cells were treated with 50 µM MG132 for 5 hours prior to harvesting. MG132 was included to minimize the degradation of AID by the proteasome [19], although it was not necessary to detect the AID/CIB1 interaction (data not shown). The cells were washed in cold PBS and resuspended in lysis buffer [25 mM HEPES, pH 7.4; 150 mM NaCl; 1 mM EDTA; 1 mM MgCl_2_; 1 mM ZnCl_2_; 10% glycerol; 1% NP40; protease inhibitors (Complete, EDTA-free, Roche)], on a rotating wheel for 1 hour at 4°C. The lysate was clarified by centrifugation at 13,000 *g* for 20 minutes and 300 µl was incubated with 2.5 µL (2.5 µg) α-myc mAb (LabVision) on a rotating wheel for 30 minutes at 4°C before the addition of 25 µL of pre-washed Protein G Sepharose (GE Healthcare). Bound complexes were washed three times with lysis buffer and once with TBS/0.05% Tween 20. Proteins were eluted off the beads with 0.1 M glycine (pH 2.5) and resolved by 10% SDS-PAGE. The proteins were transferred to PVDF membrane and blotted with a rabbit α-GFP polyclonal Ab (Invitrogen) to detect AID-GFP.

Reciprocal pulldowns were conducted by transfecting pEGFP-N3-AID or pEGFP-N3 and 6xmyc-hCIB1 as above. Lysates (300 µL) were incubated with 20 µg of RNase A, DNase I or BSA at 37°C for 20 minutes (Sigma). They were then incubated with 5 µL (5 µg) of α-GFP mAb (Clontech JL8) on a rotating wheel for 30 minutes at 4°C before the addition of 25 µL of washed Protein G Sepharose beads. The beads were washed, eluted, and transferred as above then blotted with α-myc (LabVision 9E11 mAb).

For the endogenous CIB1 pull-down experiments, HEK-293T cells were transfected as above with pAID-STZ or pEGFP-STZ. These proteins were precipitated with IgG Sepharose (GE Healthcare), removed from beads by TEV protease cleavage, processed as above to a PVDF membrane, and probed first with an α-CIB1 monoclonal antibody [53] and second with an α-Strep monoclonal antibody (Novagen). Primary antibodies were detected with α-rabbit-HRP or α-mouse-HRP secondary reagents (BioRad).

### DT40 experiments

Surface Ig-negative DT40 cells were maintained in RPMI (Hyclone) supplemented with 10% FBS (Gibco) and 50 µM 2-mercaptoethanol. Cells were subcloned by limiting dilution and grown continuously for 6 weeks. The IGC capacity was determined by measuring the percentage of cells that reverted to surface Ig-positive by staining with R-PE conjugated mouse α-chicken Ig antibody (Southern Biotechnology Associates, Inc.) and quantifying labeled cells by flow-cytometry (FACSCalibur, BD Biosciences). 8–12 independent subclones were examined for each condition and the median percentage of surface Ig-positive cells was used to assess gene conversion capacity.

CIB1-deficient DT40 was constructed by serial gene targeting [26], [35]. Targeting constructs were linearized with NotI and electroporated into 20 million cells (500 V, 25 µF; BioRad GenePulser II). Following the appropriate drug selection (0.25 µg/ml puromycin, 10 µg/ml blasticidin), single clones were isolated and expanded for screening by PCR, RT-PCR, and Southern blotting. For PCR screening, a common reverse primer 5′-CTT-TGT-GCC-TCC-CGT-TAG-AG located 3′ to the targeting arm was used in combination with forward primers designed specifically for exon 5 5′-GAT-GGC-ACC-ATC-AAC-CTC-TC, the blasticidin drug cassette 5′-GAA-GAC-CCC-AAG-GAC-TTT-CC, or the puromycin cassette 5′-CCC-CCT-GAA-CCT-GAA-ACA-TA. To confirm loss of CIB1 mRNA expression, cDNA was generated from total mRNA and subjected to RT-PCR using primers spanning intron 4 5′-GGG-ATG-ACA-GCA-TGT-CCT-TT and 5′-TGA-GCT-GCT-CCA-TCT-CAA-TG. Primers for AID expression were used to verify the presence of cDNA 5′-TGG-ACA-GCC-TCT-TGA-TG and 5′- GTC-CCA-GAG-TTT-TAA-AG. For Southern blotting, a 3′ probe was generated by PCR from DT40 genomic DNA using primers 5′-GAT-CCC-TCC-CTC-CTT-GGG-AGA and 5′-CCG-GAG-GCG-TTG-GCT-GGT-GCC.

DT40 lines stably expressing chicken CIB1 or human CIB1 were generated by electroporating linearized (BglII) p6xmyc-hCIB1 or p6xmyc-cCIB1 (250 V, 950 µF, 2 pulses; BioRad GenePulser II) into surface Ig negative DT40, selecting with 1.5 mg/ml Geneticin (Invitrogen), and screening for expression by immunoblotting. 2×10^6^ cells were lysed in NP40 lysis buffer [25 mM HEPES, pH 7.4; 150 mM NaCl; 1 mM EDTA; 1 mM MgCl_2_; 1 mM ZnCl_2_; 10% glycerol; 1% NP40; protease inhibitors (Complete, EDTA-free, Roche)] on ice for 1 hr and then clarified by centrifugation (16,100 g, 5 min). The supernatants were denatured in Laemmli loading buffer and separated by SDS-PAGE. Proteins were transferred to PVDF membrane and blotted with α-myc (LabVision 9E11 mAb) to detect transfected CIB1 or α-tubulin (Covance mAb).

AID localization was determined in DT40 by transducing cells with a lentivirus encoding AID-EGFP (above). Lentiviruses were produced by transfecting HEK-293T cells with pCSII-AID-EGFP, the VSV-G envelope vector pMDG, and the ΔNRF packaging construct encoding the HIV-1 gag, pol, rev, tat, and vpu genes as described previously [51]. DT40 cells were cultured in lentiviral supernatant for 12 hrs to enable viral transduction. After an additional 36 hrs, the cells were washed with fresh media and AID-EGFP localization was determined by fluorescence microscopy (40× objective; Deltavision).

### Mouse experiments

Experimental procedures were conducted in accordance with the University of Minnesota Institutional Animal Care and Use Committee guidelines. The CIB1-deficent mice were reported previously [23]. Heterozygous mice were bred to produce experimental wildtype and CIB1-deficient littermates, which were housed approximately 7 weeks and then used for experiments.

To quantify serum Ig levels, 50–100 µl blood was collected from mice via facial vein bleeding and allowed to clot at room temperature. After removing cells by centrifugation, serum antibody titers for isotypes IgM, IgG1, IgG2a, IgG2b, IgG3, and IgA were determined by ELISA using a Mouse Immunoglobulin Isotyping Kit (BD Pharmingen).

To determine the frequencies of *ex-vivo* CSR, resting B-cells were isolated from dissociated spleen through magnetic sorting by negative selection using an antibody cocktail against CD43 (Ly-48), CD4 (L3T4), and Ter-119 (Miltenyi Biotech). Isolated B-cells shown to be over 90% CD19 positive were then cultured for in RPMI media (Gibco) supplemented with 10% FBS plus 50 ng/ml IL-4 (R&D Systems) and 50 µg/ml LPS (Sigma). After 4 days, cells were analyzed for CSR by flow-cytometry using a α-IgG1-PE antibody (BD Biosciences).

For histology experiments, spleens were removed from euthanized animals, preserved in 10% phosphate-buffered formalin, sliced into 10 µm thick sections (Leica Instruments), stained with hematoxylin and eosin, and examined by light microscopy to identify germinal centers (Zeiss Axiovert).
